# Lawmakers Demand Answers From EPA

**DOI:** 10.1100/tsw.2000.4

**Published:** 2001-04-04

**Authors:** Nicole Ruediger

## Abstract

Washington, Oct. 6—Early this afternoon, lawmakers demanded answers from Environmental Protection Agency (EPA) Administrator Carol Browner. In a letter sent earlier today to Browner, House Science Committee Chairman F. James Sensenbrenner, Jr. (R-WI) demanded to know why her testimony and that of one of her deputies, Ramulo Diaz, at Wednesdays Science Committee hearing conflicted with actions taken yesterday by the agency.

The conflict involves the EPA's decision to reassign Rosemarie Russo, Director of the EPA Office of Research and Development (ORD) in Athens, Georgia. Last Monday, the Department of Occupational Safety and Health Administration (OSHA) issued a ruling stating that it had found "credible evidence" that the EPA had improperly retaliated against Russo after she provided testimony to the House Science Committee for an EPA oversight hearing and for allowing one of her colleagues, David Lewis, to publish a paper in the journal *Nature* which called into question EPA risk assessments.

Browner testified under oath during Wednesday's hearing, "The reassignment did not occur." However, yesterday, the Assistant General Counsel of the EPA issued a letter appealing OSHA's findings, asserting that the EPA's decision to reassign Russo was legitimate.

Sensenbrenner wrote in his letter to Browner, "The only apparent explanation is that you either intentionally misled this Committee or EPA's Office of Research and Development, Office of General Counsel and Office of Administration and Resources Management (which Mr. Diaz heads) are acting on their own without consent from you or Mr. Diaz."

The EPA has until October 10, 2000 to provide the House Science Committee with a letter explaining their actions.

An EPA spokesman told TheScientificWorld, "The agency fully hopes to satisfy Chairman Sensenbrenner's inquiry."

—Nicole Ruediger

Previously posted in NewsLab, TheScientificWorld.
© 2000 TheScientificWorld, Inc.
